# Japanese Spotted Fever in Eastern China, 2013

**DOI:** 10.3201/eid2411.170264

**Published:** 2018-11

**Authors:** Jiabin Li, Wen Hu, Ting Wu, Hong-Bin Li, Wanfu Hu, Yong Sun, Zhen Chen, Yonglin Shi, Jia Zong, Adams Latif, Linding Wang, Li Yu, Xue-Jie Yu, Bo-Yu Liu, Yan Liu

**Affiliations:** The First Affiliated Hospital of Anhui Medical University, Hefei, China (J. Li, T. Wu, H.-B. Li);; The First Affiliated Hospital of the University of Science and Technology of China, Hefei (Wen Hu);; Anhui Center for Disease Control and Prevention, Hefei (Wanfu Hu, Y. Sun, Y. Shi);; Anhui Medical University, Hefei (Z. Chen, J. Zong, A. Latif, L. Wang, L. Yu, B.-Y. Liu, Y. Liu);; Wuhan University School of Health Sciences, Wuhan, China (X.-J. Yu)

**Keywords:** *Rickettsia japonica*, spotted fever group Rickettsiae, Japanese spotted fever, *Haemaphysalis longicornis* ticks, rash, papular rash, fever, China, seroprevalence, blood chemistries, urinalysis, Anhui Province, Shandong Province, electron microscopy, phylogenetic analysis, tickborne infection, vector-borne infections, bacteria, 17-kDa protein, 16S rRNA

## Abstract

We isolated *Rickettsia japonica* from a febrile patient in Lu’an City, China, in 2013. Subsequently, we found an *R. japonica* seroprevalence of 54.8% (494/902) in the rural population of Anhui Province and an *R. japonica* prevalence in *Haemaphysalis longicornis* ticks of 0.5% (5/935). *R. japonica* and its tick vector exist in China.

Spotted fever group rickettsiae are tickborne, obligatory intracellular, gram-negative bacteria with a worldwide distribution. However, the distribution of each species of spotted fever group rickettsiae is limited to geographic areas by their specific tick vectors. Japanese spotted fever is a severe rickettsiosis caused by *Rickettsia japonica* bacterium (*1**,**2*), which has been present in Japan since 1984 and isolated from patients in other countries of Asia (e.g., South Korea, the Philippines, and Thailand) over the past decade (*3**,**4*)*.* In this study, we present information on an *R. japonica* isolate acquired from a febrile patient and *R. japonica* seroprevalence in Anhui Province in eastern China.

On August 7, 2013, a 61-year-old man from Shucheng County, Lu’an City, China, in the Dabie Mountain area of Anhui Province (Technical Appendix Figure 1) with fever and headache for 1 week was admitted into Shucheng County People’s Hospital. The patient reported several tick bites 10 days before the onset of his illness. At admission, the patient was conscious and had fever (39.0°C); he did not have jaundice, and no bleeding was found on his skin or mucosal membranes. A papular rash with papules 0.1–0.5 cm in diameter was noted all over his body (Technical Appendix Figure 2). Blood cell counts showed the patient had leukocytosis (10.34 × 10^9^ cells/L), increased neutrophils (87.5%), and a platelet count within reference range (130 × 10^9^/L). Blood chemistry testing revealed a urea nitrogen concentration of 9.12 mmol/L (reference range 2.9–8.2 mmol/L), creatinine of 0.758 mg/dL (67 μmol/L, reference range 53–106 μmol/L), C-reactive protein of 77.5 nmol/L (reference range 0.76–28.5 nmol/L), and an erythrocyte sedimentation rate of 22 mm/h (reference range 0–20 mm/h). A urine test showed a procalcitonin concentration of 0.806 ng/mL (reference range <0.15 ng/mL) and an interleukin 6 concentration of 52 pg/mL (reference range <1.8 pg/mL). The patient had rough lung breath sounds, and computed tomography showed inflammatory infiltrates in the middle right lung and lower left lung lobe, bullae on the upper left lung lobe, and emphysematous changes. The patient was suspected to have a rickettsial infection and was given minocycline and meropenem on the day of his admission. Two days later, on August 9, 2013, the patient’s fever subsided (36.2°C), and he was discharged.

A blood sample taken from the patient 1 day after admission was inoculated onto THP-1 and Vero E6 cells; after 10 days, cytopathic effect was visible by light microscopy with only the THP-1 cells. Diff-Quick (Thermo Fisher Scientific, Kalamazoo, MI, USA)–stained smears of THP-1 cells showed *Rickettsia*-like bacilli in the cytoplasm. Electron microscopy showed the bacilli localized to the cytoplasm and nucleus and had the typical ultrastructure of *Rickettsia* bacteria. This species was highly pleomorphic but mainly had dimensions 0.2 μm × 0.5–1 μm (Technical Appendix Figure 3).

We amplified and sequenced the 17-kDa protein gene, 16S rRNA gene, *ompA*, *ompB*, and gene D of *R. japonica* (GenBank accession nos. KY364904, KY484160, KY484162, KY484163, and KY488633; Technical Appendix Table). These gene sequences were 99.8%–100% homologous with the corresponding gene of an *R. japonica* isolate (GenBank accession no. AP017602.1).

Hard-body tick species *Haemaphysalis longicornis*, *H. flava*, and *Dermacentor taiwanensis* (*5**,**6*) have been reported as *R. japonica* transmission vectors. We acquired questing *H. longicornis* ticks in Shandong Province, China, in 2013 and found them positive for the *R. japonica* 17-kDa protein and 16S rRNA genes by PCR (online Technical Appendix). The percentage of *H. longicornis* ticks infected with *R. japonica* rickettsia in Shandong Province was 0.5% (5/975). The *H. longicornis* tick, which is prevalent in East China and feeds on domestic animals and small mammals, might be a major vector of *R. japonica* rickettsia in China (*7**,**8*). Phylogenic analysis of the 16S rRNA (Figure, panel A) and 17-kDa protein (Figure, panel B) genes indicated that the rickettsial isolates from the patient and *H. longicornis* tick were identical to *R. japonica* isolates and in the same clade with *R. heilongjiangensis*.

**Figure:**
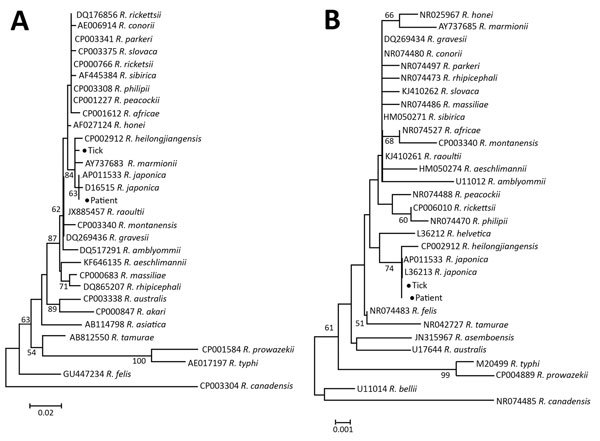
Phylogenetic analysis of *Rickettsia* isolate from patient with Japanese spotted fever in Anhui Province and isolate from *Haemaphysalis longicornis* tick in Shandong Province, China, 2013 (black dots), compared with reference isolates. Unrooted neighbor-joining trees of 16S rRNA gene (A) and 17-kDa protein gene (B) were constructed by using MEGA 5.2 (https://www.megasoftware.net/) and 1,000 bootstrap replications. Scale bars represent substitutions per nucleotide.

Examination by indirect immunofluorescence assay showed that the patient’s acute (1:80 dilution) and convalescent (1:1,280 dilution) serum samples reacted to isolated antigen of *R. japonica* bacterium. During 2013, we collected serum samples from 902 healthy persons living in rural areas of Anhui Province (Technical Appendix Figure 1) and tested them with the same assay. We found 54.8% (494/902) of serum samples positive for *R. japonica*–specific antibodies.

In summary, we detected *R. japonica* bacteria in a patient and an *H. longicornis* tick and demonstrated high *R. japonica* seroprevalence among the rural population of Anhui Province. In agreement with Lu et al.’s work in 2015 (*9*), our findings suggest that *R. japonica* might be more prevalent in China than previously thought. Physicians in China need to become aware of *R. japonica* disease presentation, so they can administer the appropriate treatment to patients with suspected *R. japonica* infections.

Technical Appendix Description of methods and primers, clinical image of patient rash, electron micrograph of *Rickettsia japonica* isolate obtained from patient, and map showing location of patient in case study and of serum sample collection for seroprevalence study.
