# Supplementary material for: Japanese Spotted Fever in Eastern China, 2013
Source: Emerg Infect Dis. 2018 Nov;24(11):2107–9. doi: 10.3201/eid2411.170264 (PMC6199993; doi:10.3201/eid2411.170264)
Supplement: Technical Appendix — Description of methods and primers, clinical image of patient rash, electron micrograph of Rickettsia japonica isolate obtained from patient, and map showing location of patient in case study and of serum sample collection for seroprevalence study. [file 17-0264-Techapp-s1.pdf]

Article DOI: https://doi.org/10.3201/eid2411.170264

Japanese Spotted Fever in Eastern China, 2013

Technical Appendix

Methods

Patient and Healthy Population

We obtained the patient's clinical data from Shucheng County People's Hospital (Anhui Province, China). We also collected 902 serum samples from healthy persons in rural areas of Anhui Province, China, during August–December 2013. All participants provided written informed consent for collection and testing of blood samples. The research protocol was approved by the human bioethics committee of Anhui Medical University.

Isolation of Pathogen

An anticoagulated acute blood sample was obtained from the patient on day 1 after hospitalization and was inoculated onto monolayers of Vero E6 cells and THP-1 cells in 12.5-cm^2 flasks. The THP-1 cells were cultured with RPMI (Roswell Park Memorial Institute) 1640 medium with 10% fetal bovine serum, and Vero E6 cells were cultured with minimum essential media with 5% fetal bovine serum. Cells were incubated at 35°C in an atmosphere of 5% carbon dioxide. A smear from each flask was stained with Diff-Quick (Thermo Fisher Scientific, Kalamazoo, MI, USA) every day and observed by using light microscopy to determine the presence of intracellular bacteria.

Serologic Testing

Serum samples from the patient and healthy persons were tested for IgG specific to *Rickettsia* bacteria by indirect immunofluorescence assay. We seeded slides with Vero E6 cells and infected cells with purified *Rickettsia* bacteria isolated during this study. Serum samples were diluted 4 times in 2-fold increments (1:80–1:1,280). A person with a reciprocal titer of ≥1:80 was considered seropositive.

Transmission Electron Microscopy

The isolated organism was cultivated in THP1 cells in a 25-cm^2 flask. When 90% of the cells were infected, the cells were harvested and incubated with a fixative solution containing 2.5% formaldehyde, 0.1% glutaraldehyde, 0.03% $CaCl_2$, and 0.03% trinitrophenol in 0.05 M cacodylate buffer, pH 7.4. After fixation, the sample was stained with 1% osmium tetroxide in 0.05 M cacodylate buffer (pH 7.2) en bloc for 20 minutes with 2% aqueous uranyl acetate at 60°C, dehydrated in a graded series of ethanol, and embedded in Poly/Bed 812 (Polysciences Inc., Warrington, PA, USA). Ultra-thin sections were cut on a UC7 ultra microtome (Leica, Wetzlar, Germany), stained with lead citrate, and examined in a Philips 201 transmission electron microscope (Eindhoven, the Netherlands) at 60 kV.

Tick Samples

Questing ticks were collected during June and July 2013 in Shandong Province, China, by flagging over vegetation. The ticks were frozen at –80°C until use. Tick species and developmental stages were identified morphologically, and the tick species was molecularly confirmed. A total of 975 ticks (540 nymphs and 435 adult ticks) were pooled into groups (containing either 20 nymphs or 5 adult ticks) and used for DNA extraction.

PCR Amplification and DNA Sequencing

DNA was extracted from cell culture materials with the QIAamp DNA Mini Kit (QIAGEN, Hilden, Germany). The DNA samples were tested by PCR with primers designed to

amplify 5 rickettsial genes: 16S rRNA gene, 17-kDa protein gene, gene D, *ompA*, and *ompB* (Technical Appendix Table). Both strands of the PCR products were sequenced and compared with those in GenBank by using Blast (https://blast.ncbi.nlm.nih.gov/Blast.cgi).

Phylogenetic Analysis

The DNA sequences of the 17-kDa protein gene and 16S rRNA gene of the *Rickettsia* species isolated in this study and DNA sequences of *Rickettsia* spp. from the GenBank database were aligned and compared by MEGA (https://www.megasoftware.net/). Phylogenetic trees were constructed by using the neighbor-joining method in MEGA 5 with 1,000 bootstrap replications.

References

1. Sekeyová Z, Fournier PE, Rehácek J, Raoult D. Characterization of a new spotted fever group rickettsia detected in *Ixodes ricinus* (Acari: *Ixodidae*) collected in Slovakia. J Med Entomol. 2000;37:707–13. PubMed http://dx.doi.org/10.1093/jmedent/37.5.707

2. Suksawat J, Xuejie Y, Hancock SI, Hegarty BC, Nilkumhang P, Breitschwerdt EB. Serologic and molecular evidence of coinfection with multiple vector-borne pathogens in dogs from Thailand. J Vet Intern Med. 2001;15:453–62. PubMed http://dx.doi.org/10.1111/j.1939-1676.2001.tb01574.x

3. Wenzel RP, Hayden FG, Gröschel DH, Salata RA, Young WS, Greenlee JE, et al. Acute febrile cerebrovasculitis: a syndrome of unknown, perhaps rickettsial, cause. Ann Intern Med. 1986;104:606–15. PubMed http://dx.doi.org/10.7326/0003-4819-104-5-606

4. Webb L, Carl M, Malloy DC, Dasch GA, Azad AF. Detection of murine typhus infection in fleas by using the polymerase chain reaction. J Clin Microbiol. 1990;28:530–4. PubMed

5. Andersson JO, Andersson SG. Genome degradation is an ongoing process in *Rickettsia*. Mol Biol Evol. 1999;16:1178–91. PubMed http://dx.doi.org/10.1093/oxfordjournals.molbev.a026208

Technical Appendix Table. Primers for amplification and sequencing of genes of *Rickettsia* spp. isolated from patient with Japanese spotted fever, Anhui Province, China, 2013

Gene	Primer name	Nucleotide sequence	Position	Reference
*omp*A	190.70f	5′-ATGGCGAATATTTCTCCAAAA-3′	70–90	(*1*)
	190.701r	5′GTTCCGTTAATGGCAGCATCT-3′	701–681	
*omp*B	M59f	5′CCGCAGGGTTGGTAACTGC-3′	M59–M41	(*2*)
	807r	5′CCTTTTAGATTACCGCCTAA-3′	807–788	
16S rRNA	fD1out-f	5′-AGAGTTTGATCCTGGCTCAG-3′	339–357	(*3*)
	357f	5′TACGGGAGGCAGCAG-3′	334–348	
	800r	5′CTACCAGGGTATCTAAT-3′	828–812	
	1050r	5′CACGAGCTGACGACA-3′	1,109–1,095	
	rP2-out-r	5′-ACGGCTACCTTGTTACGACTT-3′	1,406–1,392	
17-kDa protein	17kD-f	5′GCTCTTGCAACTTCTATGTT-3′	31–50	(*4*)
	17KD-r	5′CATTGTTCGTCAGGTTGGCG-3′	464–445	
D	D1f	5′ATGAGTAAAGACGGTAACCT-3′	1–20	(*5*)
	D928r	5′-AAGCTATTGCGTCATCTCCG-3′	928–907	

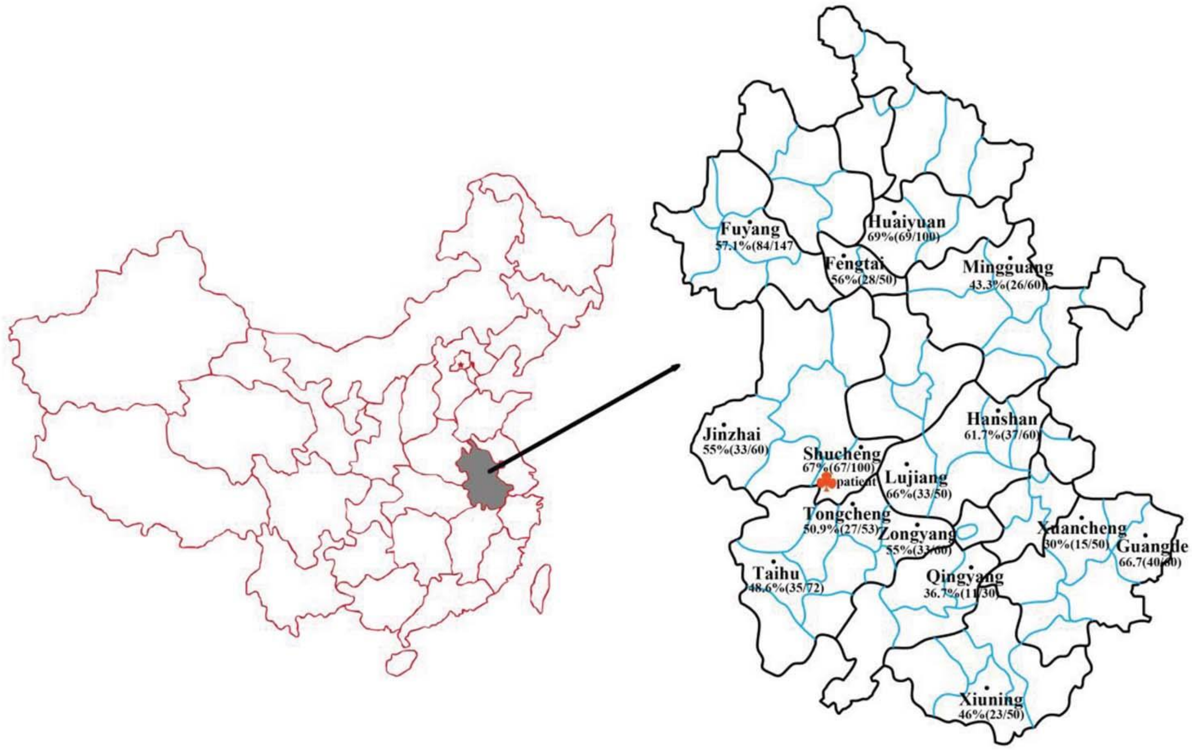

Technical Appendix Figure 1. Location of patient with Japanese spotted fever in 2013 and collection sites for serum samples from healthy persons, Anhui Province, China. The location of Anhui Province in China is indicated on the left. The location of the patient's hometown is marked with a clover. The serum samples were tested by an indirect immunofluorescence assay that included the bacteria isolated from the patient with Japanese spotted fever as the antigen. The percentage of healthy persons with antibodies specific to this pathogen and numbers of positive and total samples are indicated.

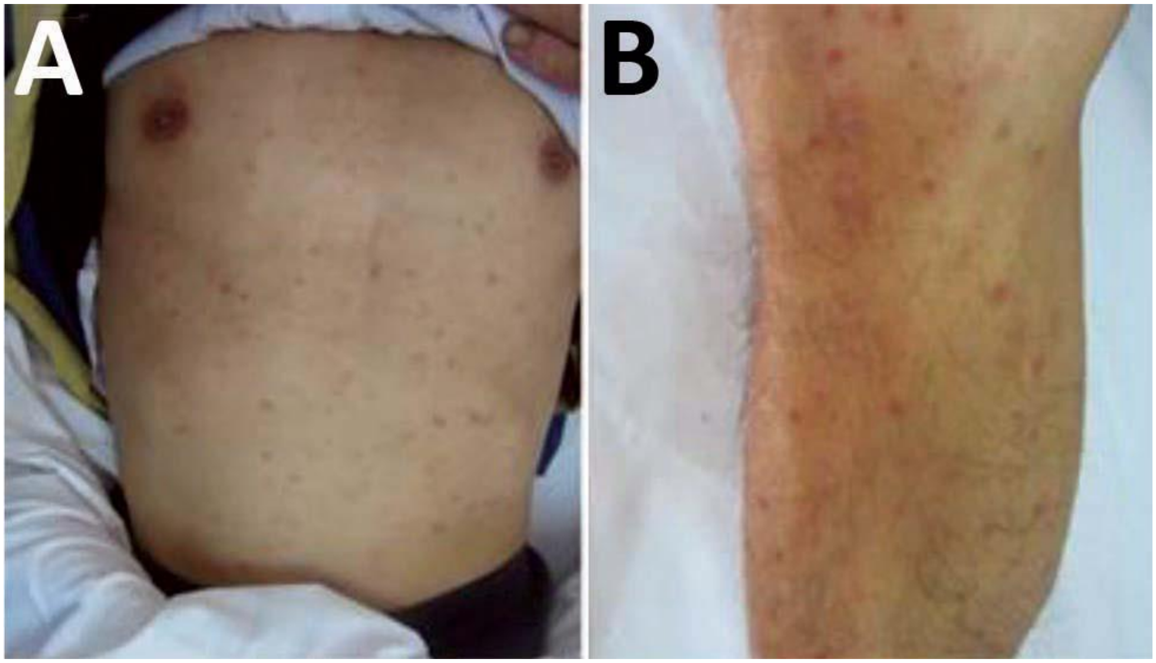

Technical Appendix Figure 2. Rash on 61-year-old-man with Japanese spotted fever, Anhui Province, China, August 2013. Papular rash on trunk (A) and leg (B) of patient.

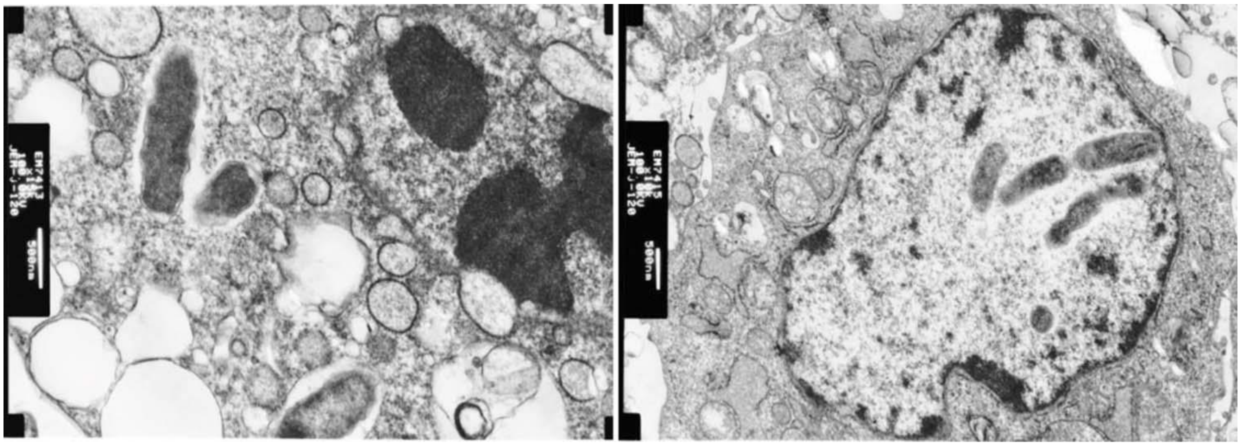

Technical Appendix Figure 3. Electron micrograph of etiologic agent isolated from blood sample of patient with Japanese spotted fever, Anhui Province, China, August 2013. Patient blood sample was collected on day 1 after hospital admission, and pathogen was cultured in THP-1 cells. The bacterium was observed in the cytoplasm and nucleus by electron microscopy. Bar represents 0.5 μm.
